# Duodenal gastrointestinal stromal tumor: A case report

**DOI:** 10.1016/j.amsu.2022.104574

**Published:** 2022-09-08

**Authors:** Saroj Kumar Yadav, Himal Bikram Bhattarai, Ashes Rijal, Anish Shrestha, Sangam Shah, Ayusha Subedi, Binita Kumari Yadav, Aakash Acharya, Rojee Khatri, Gareema Kadel

**Affiliations:** a Department of General Surgery, Koshi Hospital, Biratnagar, Nepal; b Gandaki Medical College, Pokhara, Nepal; c Tribhuvan University, Institute of Medicine, Maharajgunj, 44600, Nepal; d Manmohan Memorial and Community Hospital, Jhapa, Nepal; e Greencross Hospital, Biratnagar, Nepal; f College of Medical Sciences, Bharatpur, Nepal; g Grande International Hospital, Kathmandu, Nepal

**Keywords:** Duodenal GIST, GI bleed, Imatinib

## Abstract

**Introduction:**

Gastrointestinal tumor (GIT) is an uncommon gastrointestinal tumor most commonly arising in the stomach. Duodenum is an uncommon site accounting for only about 3–5% of cases.

**Case presentation:**

In this case report, we present a case of high-risk duodenal GIST and review its management strategies.

**Discussion:**

An abdominal mass and gastrointestinal (GI) bleeding are its usual presentation, however it may be lost among the long list of differentials of an abdominal mass and GI bleeding, if a high index of suspicion is not maintained. Surgery, with or without tyrosine kinase inhibitors like imatinib mesylate, has been the cornerstone in management of GIST.

**Conclusion:**

This case underlined the importance of duodenal GIST as a cause of GI bleed and abdominal mass as well as shone light upon historical developments, current updates and managements of GISTs.

## Highlights

• Gastrointestinal tumor (GIT) is an uncommon gastrointestinal tumor most commonly arising in the stomach.
• Duodenal GIST as a cause of GI bleed and abdominal mass.
• Surgery, with or without tyrosine kinase inhibitors like imatinib mesylate, has been the cornerstone in management of GIST.
• Here, we presented a rare case of duodenal GIST which should be considered a differential when considering etiologies for GI bleed.

## 1 Introduction

Gastrointestinal Stromal Tumor is an uncommon cause of primary gastrointestinal neoplasm accounting for less than 1% of all GI tumors [1]. The median age at diagnosis is about 60 yrs [2].In 1983, they were originally designated as an unclassified group of GI tumors of smooth muscle or neurogenic origin. However, later were discovered to be originating from interstitial cells of Cajal [3]. This was primarily after its analysis with immunohistochemical techniques-while GISTs are CD 117 positive, tumors arising from smooth muscle cells such as leiomyomas are not [4].GIST is most often located in the stomach (55.6%) and jejunum/ileum (31.8%) [2]. Duodenal GISTs account for around an estimate of 3–5% cases among gastrointestinal GISTs [5,6]. Based on its location and disease spread GIST can present with a wide array of clinical symptoms ranging from subtle dyspepsia/no symptoms to intestinal obstruction and hemorrhage. In fact, only around 69% of cases were symptomatic [7]. Here, we present a case of symptomatic duodenal GIST who presented to our center with a palpable mass in the abdomen and a history of gastrointestinal bleed resulting in severe anemia. The case has been described as per SCARE 2020 criteria [8].

## 2 Case presentation

A 64-year-old male from eastern part of Nepal, presented in the outpatient department of our hospital with the complaints of black colored stool for 1 month. In addition to this, he also had weakness and exertional dyspnea. There was no history of a significant loss of weight nor a history of change in his appetite. Physical examination revealed pallor and an ill-defined abdominal mass occupying the right hypochondriac and the right lumbar regions. Lymph nodes assessment were unremarkable during the physical examination. On further review, his blood investigations showed a hemoglobin level of 5.6 gm/dl. He received blood transfusion in the hospital and was admitted for further evaluation of his symptoms.

**Fig. 1:**
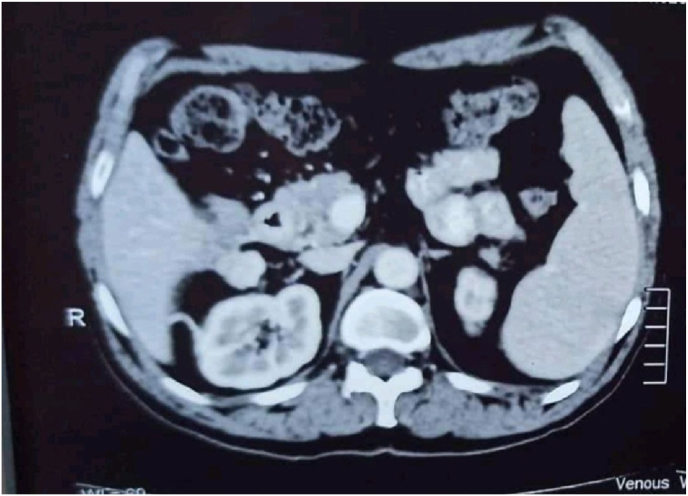
CT abdomen showing encapsulated mass arising from 2nd part of duodenum (axial section).

**Fig. 2:**
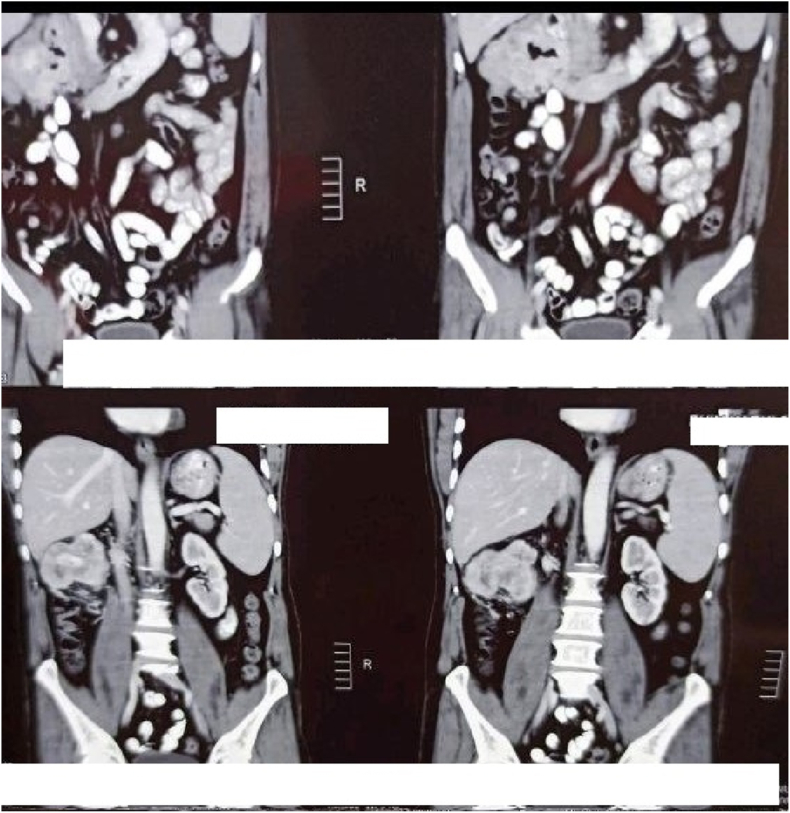
CT abdomen showing encapsulated mass extending to inferior surface of liver (coronal section).

**Fig. 3:**
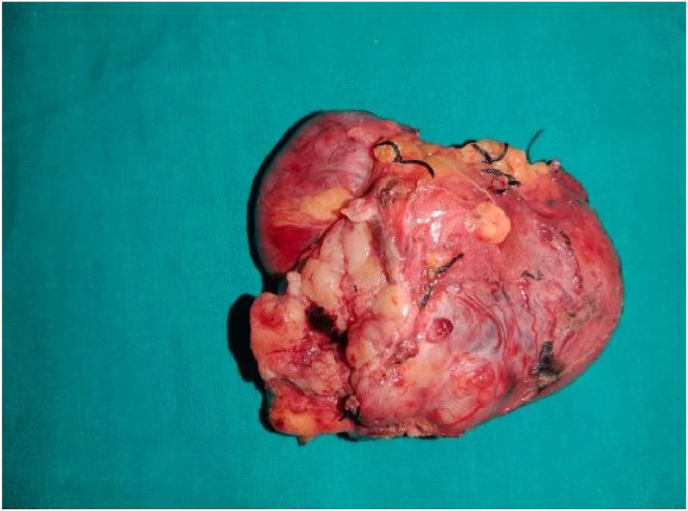
Gross specimen showing encapsulated mass arising from 2nd part of duodenum.

**Fig. 4:**
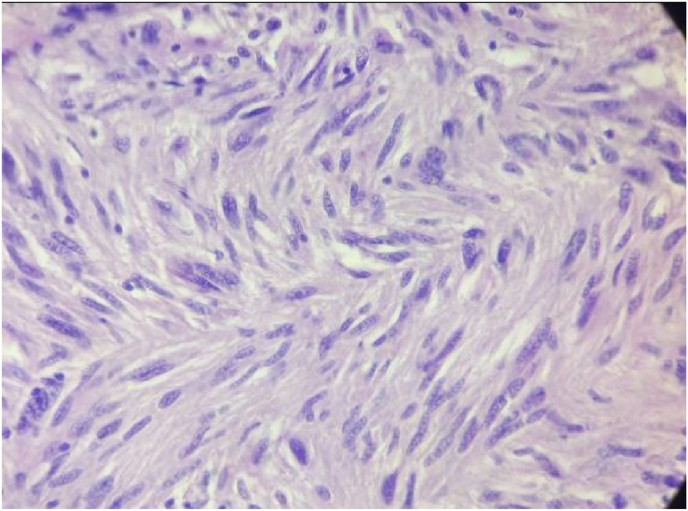
Histologic picture of the specimen showing tumor cells with elongated nuclei and inconspicuous nucleoli and faint eosinophilic cytoplasm (magnified).

**Fig. 5:**
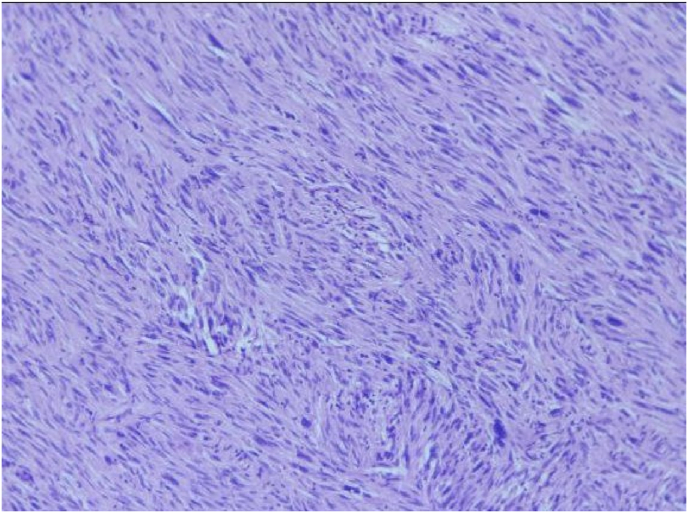
Histologic picture of the specimen showing spindle shaped tumor cells (magnified).

An initial assessment via. an upper gastrointestinal endoscopy revealed an ulcer of 1*0.5 cm located on the lateral aspect of the second part of duodenum following which, a Contrast Enhanced Computed Tomography (CECT) scan of the abdomen was ordered. Report of the CT scan showed a well encapsulated lobulated mass measuring 11*9 cm arising from the second part of the duodenum compressing on the liver and the right kidney and abutting IVC as shown in Fig. 1 and Fig. 2. After a thorough discussion with the patient, a laparotomy was planned. During the procedure, an en bloc excision of the mass with sleeve resection of the second part of the duodenum was performed. Primary repair of the duodenum with a feeding jejunostomy was also done in the same setting. Intra-operatively a 12*9 cm (Fig. 3), well defined lump arising from the second part of the duodenum extending superiorly to the inferior surface of the liver maintaining fat planes with segment V and VI of the liver were seen. Inferiorly, the mass extended till the transverse colon and posteriorly compressed the right kidney with loss of fat planes. Rest of the viscera was normal. There was no evidence of ascites or metastasis. The specimen was sent for histopathological examination which confirmed the diagnosis of a low grade duodenal gastrointestinal stromal tumor, spindle cell type (Fig. 4&5). The gross and histopathological picture are shown in Fig. 3, Fig. 4, Fig. 5. Immunohistochemistry was positive for CD 117. Imatinib was started for 3–5 years. He was discharged satisfactorily after a month of his surgery and kept on regular follow-up schedules. Reviews after a month and a year later were reassuring, and he is currently recovering well.

## 3 Discussion

In this case, the male presented with an abdominal mass and a history gastrointestinal bleed, both of which are common presentations of GISTs [1]. But when these symptoms are present in isolation, GISTs fall fairly lower in the list of differential diagnosis, thus demanding a high index of suspicion in part of the treating physician. Similarly, 10–25% of cases present with metastatic spread during the initial presentation [9]. Mitotic activity and tumor size are the major prognostic factors dictating reoccurrence or metastatic growth as exemplified in Table 2 below. Duodenal GISTs with a small size (≤2 cm) and a low mitotic activity (<5 per 50 hpf) have excellent prognosis. Metastasis can already be expected for tumors of size 2–5 cm even with a low mitotic activity. A high mitotic activity and a large tumor size(>5cm) often correlates with the worst outcomes. This is in contrast to gastric GISTs, which have better prognosis compared to small intestinal GISTs with similar size and mitotic activity [10]. The findings of a study done by Miettinen et al. is summarized in Table 1 which shows duodenal GISTs to have a higher chance of progression when compared to their gastric counterparts matched for their size and mitotic index. In our case, the size of the tumor was exceptionally large (12*9cm) placing him in the high-risk category of Fletcher classification as shown in Table 2, even though the mitotic activity of the tumor was reported to be low. One would expect the possibility of a metastatic disease with such a large tumor; however, he was fortunate to have no metastatic growth during his initial assessment. Given the high risk of his disease, regular follow-up visits are warranted to detect recurrences with starting of imatinib therapy with a usual starting dose of 400 mg/day as shown by the EORTC 62005 trial [11].

**Table 1:** Rates of metastases or tumor related deaths in GISTs of stomach and small intestine by tumor grouped by mitotic rate and tumor size. Miettinen and Lasota 2006(10).

	Tumor parameters		% Of patients with progressive disease during long term follow up and characterization of risk for metastases			
Group	Size	Mitotic rate	Gastric GIST	Jejunal and ileal GISTs	Duodenal GISTs	Rectal GISTs
1	≤2 cm	≤5 per 50 HPF	0 none	0 none	0 none	0 none
2	>2 ≤5cm	≤5 per 50 HPF	1.9 very low	4.3 low	8.3 low	8.5 low
3a	>5 ≤ 10cm	≤5 per 50 HPF	3.6 low	24 moderate		
3b	>10cm	≤5 per 50 HPF	12 moderate	52 high	34 high €	57¥high€
4	≤2cm	>5 per 50 HPF	0¥	50¥		54 high
5	>2cm ≤ 5cm	>5 per 50 HPF	16 moderate	73 high	50 high	52 high
6a	>5 ≤ 10cm	>5 per 50 HPF	55 high	85 high		
6b	>10cm	>5 per 50 HPF	86 high	90 high	86 high€	71 high€

¥: denotes tumor with very small no of cases.

€: groups 3a and 3b or 6a and 6b are combined in duodenal and rectal GISTs because of small number of cases.

**Table 2:** Risk assessment for recurrence and metastasis. Adapted from Fletcher et al., 2002 [17].

Risk category	Size	Mitotic Count
Very low risk	<2cm	<5/50 HPF
Low risk	2–5 cm	<5/50 HPF
Intermediate risk	<5 cm	6-10/50 HPF
	5–10 cm	<5/50 HPF
High risk	>10cm	Any
	Any size	>10/50 HPF
	>5cm	>5/50 HPF

Historically, GISTs have perplexed physicians for decades. The inability to distinguish them from other subsets of mesenchymal tumor and delineating its origin had halted a better treatment plan. But, advancement in the understanding of its pathobiology and genetic events relating to its development has created a paradigm shift in understanding GISTs. GISTs are immunohistochemically KIT(CD 117) positive tumors which differentiates it from tumors arising from smooth muscle cells like leiomyoma [12]. KIT, is a *trans*-membrane growth factor receptor also found in interstitial cells of Cajal. Along with this, CD 34 positivity [13] and the ability of Cajal cells to differentiate into smooth muscle cells following blockade of KIT signaling pathway reconciled the theory of GIST originating from interstitial cells of Cajal [14]. Imatinib, a tyrosine kinase inhibitor, has since found its role in management of GISTs. Various researches are being done on an ideal tumor marker for the detection of GIST. Among them, DOG1 seems to be one of the most promising tumor markers [15].

For a locally resectable non metastatic tumor as ours, surgery, especially an en bloc resection is the treatment of choice. The goal is complete gross resection with an intact pseudocapsule and a negative microscopic margin [4].In addition to surgery, the decision to start adjuvant chemotherapy with imatinib is based on the risk of recurrence as described above. However, this classification system fails to address location as a prognostic factor. When considering relapse free survival, RCTs such as EORTC 62024 by Casali et al. have supported the efficacy of imatinib even among intermediate risk patient groups [16]. A 400–600 mg is generally considered as a good starting dose. It is usually continued for at least 3 years post-surgery.

Literature reveals that recurrence primarily depends on the size of tumor rather than negative margins. Such recurrences occur predominantly intra-abdominally and involve the original tumor site [18]. The risk of recurrence is highest after surgery. This risk is decreased in patients who are being treated with adjuvant imatinib therapy [19]. However, their risk of recurrent GIST increases substantially during the first few years after the discontinuation of adjuvant imatinib therapy [20]. Thus, for high-risk GIST patients treated with adjuvant therapy, follow-up imaging with a CT or an MRI scan may be done at 6-month intervals during the treatment, every 3–4 months during the first 2 years after adjuvant therapy has been stopped, and then once every 6–12 months for up to 10 years after surgery [21].

## 4 Conclusion

GISTs present as a neglected and a rare cause of GI tumor, the true prevalence of which is estimated to be far greater than that is explored. Here, we presented a rare case of duodenal GIST which should be considered a differential when considering etiologies for GI bleed. Advances in histochemical, pathobiology, molecular genetics has considerably expanded our understanding of GIST and has aided the introduction of novel treatment options such as imatinib which demands further exploration into the subject.

## Provenance and peer review

Not commissioned, externally peer-reviewed.

## Ethical approval

None.

## Sources of funding

None.

## Author contribution

SS, AR, and AS wrote the original manuscript, reviewed, and edited the original manuscript. SKY, HBB, AR, AS, SS, AS, BKY, AA, RK, and GK reviewed and edited the original manuscript.

## Consent

Written informed consent was obtained from the patient for publication of this case report and accompanying images. A copy of the written consent is available for review by the Editor-in-Chief of this journal on request.

## Research registration

Name of the registry: None
Unique Identifying number or registration ID: None
Hyperlink to your specific registration (must be publicly accessible and will be checked):

## Guarantor

Dr. Himal Bikram Bhattarai.

## Declaration of competing interest

The authors declare that they have no known competing financial interests or personal relationships that could have appeared to influence the work reported in this paper.
